# EXCESS WEIGHT AND GASTROINTESTINAL SYMPTOMS IN A GROUP OF AUTISTIC CHILDREN

**DOI:** 10.1590/1984-0462/2020/38/2019080

**Published:** 2020-03-20

**Authors:** Dayane Verissimo da Silva, Poliana Novais Moreira Santos, Danielle Alice Vieira da Silva

**Affiliations:** a Centro Universitário Tiradentes, Maceió, AL, Brazil.; b Universidade Federal de Alagoas, Maceió, AL, Brazil.

**Keywords:** Autistic disorder, Nutritional status, Obesity, Dysbiosis, Transtorno autístico, Estado nutricional, Obesidade, Disbiose

## Abstract

**Objective:**

To evaluate the nutritional status and gastrointestinal changes in children with autism spectrum disorder (ASD).

**Methods:**

Cross-sectional, descriptive analysis of 39 children with ASD aged between three and ten years old, registered in the participating association. Nutritional status was evaluated by body mass index/age and weight/age, according to the guidelines from the World Health Organization. In order to investigate whether gastrointestinal alterations occurred, the interviewees answered a questionnaire about the presence of these symptoms within the last 30 days. In order to evaluate food consumption, a 24-hour recall questionnaire was applied and the food reported were grouped as: gluten sources, casein and ultra-processed sources. For the statistical analysis, Epi-Info software version 7.2 was used. Multivariate logistic regression analysis was performed to evaluate the variables associated with gastrointestinal alterations.

**Results:**

There was a high prevalence of overweight children with autism spectrum disorder (64.1%). No child was underweight. Thirty-four children (84.2%) had gastrointestinal symptoms. Consumption of gluten was associated with gastrointestinal symptoms (β=0.38; 95%CI 0.07-0.75; p=0.02).

**Conclusions:**

The high prevalence of being overweight should be considered during the follow-up visits of children with ASD. The influence of gluten consumption on the presence of gastrointestinal symptoms was observed in this study, and the causes involved in these alterations need to be further investigated.

## RESUMO

**Objetivo:**

Avaliar o estado nutricional e a presença de alterações gastrintestinais em crianças com transtorno do espectro autista.

**Métodos:**

Estudo transversal, descritivo, composto por 39 crianças autistas com idades entre três e dez anos, cadastradas na associação participante. O estado nutricional foi analisado a partir do índice de massa corporal/idade e do peso/idade, tendo como referências as curvas da Organização Mundial da Saúde. Para investigação das alterações gastrintestinais, o entrevistado respondeu sobre a presença de alterações nos últimos 30 dias. Na avaliação do consumo alimentar foi aplicado um recordatório de 24 horas e os alimentos listados foram categorizados em: fontes de glúten, fontes de caseína e ultraprocessados. A análise estatística utilizou o *software* Epi-Info, versão 7.2. Foi realizada a análise de regressão logística multivariada para avaliar os fatores associados às alterações gastrintestinais.

**Resultados:**

Observou-se alta prevalência de excesso de peso nas crianças com transtorno do espectro autista (64,1%), não sendo registrada nenhuma criança com déficit de peso. Um total de 34 crianças (84,2%) apresentava alterações gastrintestinais. O consumo de glúten esteve associado às manifestações gastrintestinais (β=0,38; IC95% 0,07-0,75; p=0,02).

**Conclusões:**

A elevada prevalência do excesso de peso deve ser tratada com maior atenção em crianças com transtorno do espectro autista. Foi observada a influência do consumo de glúten no aparecimento das alterações gastrintestinais, sendo necessário que as causas envolvidas nessas alterações sejam mais bem investigadas.

## INTRODUCTION

Autism is a global developmental disorder (also called autism spectrum disorder - ASD) and is characterized by persistent defecits in social communication, whether it be verbal and/or nonverbal language, and in skills to develop, maintain and understand relationships. In addition to deficits with regard to social communication, those with autism have stereotyped, repetitive behavior and a narrow range of interests. The causes of ASD are not yet clearly identified and the understanding of its pathophysiology is complex.^1^

The prevalence of ASD has increased greatly in recent years, reaching the scale of a worldwide epidemic, but no central cause has been defined and the interventions applied still require further studies to confirm its effectiveness.^2^ In Brazil, there is no research on the prevalence of the disorder nationwide, however, according to data from the Center for Disease Control and Prevention (CDC), there is now one case of autism for every 110 people. Thus, it is estimated that in Brazil, with its 200 million inhabitants, there are about two million autistic people.^3^

Despite the complex etiopathogenesis of this disease, the current literature has already established that its development is linked to a series of genetic, metabolic and environmental factors that, when connected, become a kind of trigger, sparking the disease.^4^ ^,^ ^5^ Among the factors involved in the genesis of ASD, some nutritional variables have been studied, such as vitamin D deficiency and intestinal dysbiosis. ^5^ ^,^ ^6^ ^,^ ^7^

Although they are not included in the set of behavioral changes characteristic of autism, inadequate manifestations related directly or indirectly to food are also present in 30 to 90% of the cases, the most common being food selectivity and gastrointestinal changes (constipation, diarrhea, pain abdominal disease, inflammatory bowel disease, celiac disease, food intolerance).^6^ ^,^ ^8^

Given the various changes already mentioned, the public affected by ASD is at high risk of developing nutritional problems, both losing weight and gaining weight, which causes greater damage to the health of individuals already plagued with so many changes.^8^ ^,^ ^9^ Thus, the present study aimed to evaluate the nutritional status and the presence of gastrointestinal alterations in autistic children assisted by a support group association in the municipality of Maceió, Alagoas.

## METHOD

Descriptive cross-sectional study conducted with children between three and ten years old, who have ASD and who are enrolled in an autistic support group association in Maceió, Alagoas.

The institution offers assistance to 140 children and adolescents, of which 49 were in the eligible age group. Ten children with autism that was secondary to congenital anomalies and genetic syndromes such as Down syndrome, muscular dystrophy and tuberous sclerosis were excluded because of the known association of these diagnoses with gastrointestinal disorders. The final sample consisted of 39 children.

The research was approved by the Research Ethics Committee of the Centro Universitário Tiradentes under report No. 2.785.018. Data was collected by trained researchers in 2018 after the children’s legal guardians had signed a free and informed consent form.

The interview took place in a secluded place in order to maintain the patient’s integrity and limiting his or her exposure. A semi-structured questionnaire was applied, which contained identification, socioeconomic, perinatal, clinical and nutritional history, and anthropometric data. To evaluate gastrointestinal changes, the respondent answered questions regarding the occurrence of diarrhea, constipation, bloating, gas, nausea, vomiting and gastroesophageal reflux in the previous 30 days prior to the survey. In the evaluation of food intake, the respondent was asked to remember the foods the patient had eaten in the past 24 hours, and the foods listed were categorized into: gluten sources, casein sources, and ultra-processed foods. One particular food could be allocated into more than one category.

An anthropometric evaluation was performed by measuring body weight, using a digital scale, with the patient wearing light clothes and no shoes. He or she was positioned in the center of the scale platform. Height was obtained through a portable stadiometer, with the patient standing upright, arms extended along the body, feet together, and barefoot. Body mass index/age (BMI/age) and weight/age were calculated to diagnose if the patient was overweight. The classification of nutritional status was expressed as a Z score, adopting the cutoff points established by the World Health Organization (WHO).^10^ For BMI/age, the following cutoff points were adopted for categorizing the results: weight deficit, ≤-2 Z-score values; suitable weight,> -2 to <1 Z score values; overweight, represented here by the sum of being overweight and obese, with a value ≥1 Z-score value. For the weight/age index, children with ≥-3 to <-2 Z-score values were considered to be underweight; suitable weight ≥-2 to <2 Z score values; overweight: ≥ +2 Z-score values. The transformation of anthropometric values (stature/height and weight) into Z-scores of the assessed indices was performed using the Anthro-2007 program (WHO Antro-2007, Geneva, Switzerland). The WHO growth curve set was used as a benchmark and compared to the growth charts of the study group.^11^

Data were tabulated and entered twice into Excel^®^ 2010. Then, a statistical analysis was performed with the help of Epi-Info software, version 7.2 (CDC, Atlanta, USA). To describe the characteristics regarding sex, age, income and nutritional status, absolute and relative frequencies were used. Logistic regression analysis was used to assess factors associated with gastrointestinal changes. The variables with p <0.20 in the bivariate analysis, obtained by the chi-square test, were included in the multivariate analysis one by one, and increasing according to their statistical significance. The significance level was set at p<0.05 and the confidence interval at 95%.

## RESULTS

The sample was predominantly male (84.62%), with ages ranging from seven to ten years old (61.54%). It was also found that most families lived with a family income of less than one minimum wage (Table 1).

**Table 1:** Characterization of children with autism spectrum disorder from three to ten years old, according to sex, age and family income.

Variables	Frequency n=39	%
Sex		
Female	6	15.4
Male	33	84.6
Age		
3 to 6 years	15	38.5
7 to 10 years	24	61.5
Family income		
≤1 minimum wage	25	64.1
>1 minimum wage	14	35.9

**Figure 1:**
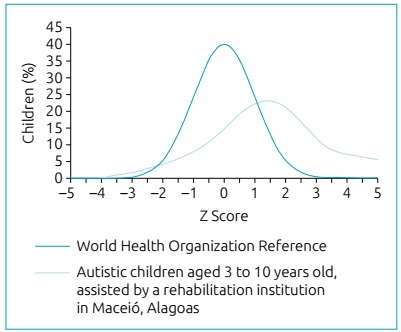
Comparison of the growth curve of autistic children aged three to ten years with the World Health Organization curve, according to body mass ­index/­age, 2018.

**Figure 2:**
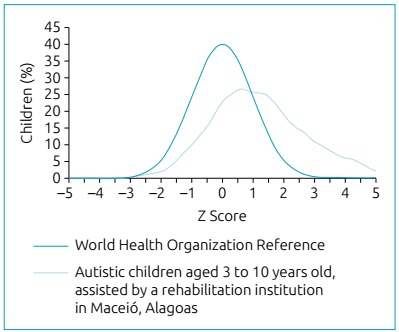
Comparison of the growth curve of autistic children aged three to ten years old with the World Health Organization curve, according to weight/age, 2018.

Regarding the classification of children’s nutritional status according to the growth pattern established by the WHO in 2007,^10^ it was observed that more than one third of the evaluated individuals were overweight (Table 2). The children in the present study generally presented higher Z-score values, according to BMI/age and weight/age, when compared to the reference curves used, which are shown graphically by the deviation of the right curve, suggesting a tendency to be overweight in the analyzed categories (Figures 1 and 2). It is worth noting that no child was underweight according to the adopted parameters.

**Table 2:** Classification of nutritional status of children with autistic spectrum disorder from three to ten years old, based on the body mass index by age and weight/age.

Variables	Frequency n=39	%
BMI/age		
Adequate	14	35.9
Overweight	25	64.1
Weight/Age		
Adequate	26	66.7
Overweight	13	33.3

BMI: body mass index.

In the analysis of food consumption and gastrointestinal changes (Table 3), it was observed that almost all of the children who consumed gluten, casein and ultra-processed foods had some gastrointestinal changes, such as reflux, gas, distension, diarrhea and constipation (n =34). However, in the logistic regression analysis adjusted for the food intake variables (gluten consumption, casein consumption and ultra-processed foods), it was observed that only gluten consumption was associated with gastrointestinal manifestations (β = 0.38; 95%CI 0.07-0.75; p=0.02).

**Table 3:** Logistic regression analysis for factors associated with gastrointestinal symptoms in autistic children aged three to ten years old.

Food consumption	Gastrointestinal symptoms (n=34)			
	Crude analysis n (%)	Adjusted analysis		
		β	95%CI	p-value
Gluten				
Yes	32 (91.3)	0.38	0.07-0.75	0.02
No	2 (9.7)			
Casein^a^				
Yes	33 (89.2)	0.26	-0.96-0.88	0.11
No	1 (10.8)			
Ultra-processed				
Yes	30 (88.2)	0.08	-0.25-0.41	0.2
No	4 (11.8)			

95%CI: 95% confidence interval; ^a^dairy products and its derivatives, whole milk, 2% milk, skim milk, and lactose-free milk.

## DISCUSSION

Autism has been showing a considerably high prevalence. It is characterized by persistent deficits with regards to social communication and stereotyped behavior, which also extend to eating habits, causing nutritional disorders.^8^ Nutritional monitoring is an important tool in these children, as it provides support for better assessments, intervention and monitoring. ^12^

Among the children evaluated, most were male, however there is still no evidence to explain the relationship between sex and occurrence of the disease. Similar to this study, Morales et al. state that the incidence of autism is four times more common in boys than in girls.^13^ Regarding nutritional status, it can be observed that no child was underweight according to the indexes evaluated. Generally, the prevalence of malnutrition in ASD occurs in children with more severe degrees of the disorder, which can be explained by their nutritional deficiencies, since most have a monotonous and inadequate diet with regard to most micronutrients.^14^ In contrast, it was possible to identify a significant number of overweight children, which is already identified as a serious public health problem in non-autistic children.^15^ These results are similar to those presented by Zheng et al. and Cria et al., which indicate that children with ASD may have a higher prevalence of being overweight and obese when compared to children with typical development. ^16^ ^,^ ^17^

According to some authors, among the risk factors that may contribute to the increased prevalence of excess weight and obesity in children with ASD, the higher food selectivity of these patients favors the increase in the consumption of snacks and highly caloric foods due to their higher palatability, thus leading to excessive weight gain.^18^ ^,^ ^19^ In addition, a relationship has been observed between pharmacological therapies, disordered sleep and weight gain in individuals with the disorder.^20^ In individuals with ASD, being overweight and obese, in addition to constituting a risk factor for cardiovascular disorders, may contribute to the worsening of social isolation, due to the individual and also society’s lack of acceptance of their body image.^21^

It is worth noting that the present study also points to an expressive consumption of ultra-processed foods, which the healthy eating guide advises against for the Brazilian population. ^22^ According to the literature, there is a strong preference for starches, processed and ultra-processed foods, in conjunction with a rejection of fruits, vegetables or proteins in children with ASD, which may contribute not only to weight gain but also to the emergence of other non-communicable chronic diseases.^23^

Like ultra-processed foods, dairy products and cereals are widely consumed by autistic children. It is suggested that the consumption of these foods may contribute to the appearance of gastrointestinal changes. The main gastrointestinal problems in children with ASD are: chronic constipation, diarrhea, abdominal pain and gastrointestinal inflammation. ^19^

The current theory of advocating for the exclusion of gluten and casein from the diet is based on findings that claim that the consumption of these proteins alters intestinal permeability because of an inflammatory reaction not yet well described. Having a cow’s milk protein allergy and celiac disease is common in these patients.^24^ ^,^ ^25^ However, the Brazilian Society of Pediatrics emphasizes that an exclusion diet should not be done as a prophylactic measure for intestinal disorders, but only in cases where an allergy is confirmed.^26^

Given this scenario of the high prevalence of gastrointestinal alterations, recent studies have raised evidence associating the relationship between intestinal dysbiosis and gastrointestinal and neurological alterations in children with ASD.^7^ ^,^ ^27^ Despite the scarcity of evidence correlating central nervous system (CNS) diseases and behavioral disorders with the intestinal microbiota, Lach et al. claim that the imbalance in the composition and diversity of this environment in childhood may favor the development and worsening of CNS diseases, as well as alter cognitive functions and sociability. ^28^

In the context of preventing or ameliorating dysbiosis, some protective factors are already described as facilitating the development of a healthy microbiota, such as the practice of exclusive breastfeeding, normal birth and full-term birth. However, there was no association between these variables and a lower frequency of gastrointestinal changes in the sample studied. According to Berding and Donovan, children who are fed artificial milk have a higher risk of developing impaired cognitive communication. In contrast, maintaining breastfeeding contributes to better CNS development and also brings additional benefit at the end of the first and second years of life, as its presence in the intestinal lumen stimulates mucosal development and activity of the lactase enzyme, thus preventing gastrointestinal changes.^2^ Despite the various theories that permeate the influence of food on gastrointestinal changes, in the present study food consumption had no influence on such manifestations.

Given the above, it is concluded that, in this population, the issue of being overweight manifests itself as a relevant problem and should be treated with greater attention, especially since it is a group of people that is more vulnerable to some complications. Furthermore, we understand that nutritional changes favor the risk of acquiring other diseases. It was also evidenced that gluten intake was associated with a greater onset of gastrointestinal changes, and more comprehensive research is recommended to clarify the causal relationship between food intake and ASD.
